# Toward a molecular understanding of adaptive immunity: a chronology – part II

**DOI:** 10.3389/fimmu.2012.00364

**Published:** 2012-11-29

**Authors:** Kendall A. Smith

**Affiliations:** The Division of Immunology, Department of Medicine, Weill Medical College, Cornell UniversityNew York, NY, USA

**Keywords:** monoclonal T cells, monoclonal antibodies, interleukins (IL-1 and IL-2), IL-2 receptor, TCR complex (T3, T4/8, Ti), α-chains, β-chains

## Abstract

By 1980 it was obvious that to more fully understand adaptive immunity, one needed to somehow reduce the tremendous complexity of antigen recognition by T cell populations. Thus, there were two developments that resulted in a paradigm shift in immunology, one being the generation of monoclonal antibodies (MoAbs), and the other the development of monoclonal functional antigen-specific T cell lines. For the first time, the cellular reagents became available to ask new questions as to how individual cells comprising the complex cell populations recognize and respond to changes in their molecular environments. The first successful generation of monoclonal T cells depended upon the understanding that antigen renders cells responsive to the antigen non-specific T cell growth factor that came to be termed interleukin-2 (IL-2), which could then be used in propagating large numbers of the progeny of single cells, which in turn could then be used for molecular analyses. Monoclonal functional human T cells were used to immunize mice to generate clone-specific (clonotypic) MoAbs, which then permitted the first biochemical characterizations of the antigen recognition elements of the T cell antigen receptor (TCR) complex. Moreover, the use of monoclonal cytolytic and helper/inducer human T cell clones essentially proved that the T cell-specific molecules T4 (CD4) and T8 (CD8) functioned as accessory molecules in antigen recognition by defining MHC class II or class I restriction respectively. As well, the expression of the T3 (CD3) molecules, found to be common to all T cells, were shown further to be obligatory for functional antigen-specific T cell signaling. The monoclonal IL-2-dependent T cells were also instrumental in the isolation and purification of the IL-2 molecule to homogeneity, the first interleukin molecule to be identified and characterized. These advances then led to the generation of pure radiolabeled IL-2 molecules that were used to identify the first interleukin cellular receptors, and as well the generation of the first MoAbs reactive with both IL-2 and IL-2 receptors. All of these advances led subsequently to the isolation of the first cDNA clones recognizing one of the two chains comprising the T cell antigen recognition elements (β-chain), as well cDNA clones encoding IL-2. Accordingly, armed with all of these unique cellular and molecular reagents, it was possible to determine that antigen triggering of the TCR complex initiates IL-2 production and IL-2 receptor expression, which in turn initiate the T cell clonal proliferative expansion, envisioned by Burnet in his formulation of the clonal selection theory. Thus, adaptive immunity receives antigen-specific activation signals from the environment and turns them into antigen non-specific endogenous action signals.

## MURINE MONOCLONAL CYTOLYTIC T CELLS

To move beyond descriptive T cell biology, it was necessary to reduce the tremendous complexity and heterogeneity of T cell populations to the progeny of a single T cell, so that it would be possible to fulfill Burnet’s prediction that, “*Only by the use of a pure clone technique of tissue culture, which allows mesenchymal cells to retain full functional activity, would we be likely to find an answer*” (to the clonal selection hypothesis; Burnet, 1959). Thus, following the lead of those who had employed repetitive alloantigen stimulation to generate long-term cultures of T cells, Fathman and Hengartner (1978) attempted to use repetitive mixed leukocyte cultures (MLC) to develop T cell clones. Unfortunately, their cultured cells lost their antigen-specific cytolytic capacities so that they could not demonstrate clonality. Nabholz et al. (1978) also tried to generate cytolytic clones of alloreactive cells using colony formation in soft agar. However, they too could not demonstrate monoclonal cytolytic function, and both groups had no way to expand any cells that they had isolated beyond repetitive MLC.

Because our long-term cytotoxic T lymphocyte lines (CTLL) had been generated against allogeneic leukemia cells, and had demonstrable alloantigen cytolytic specificity as well as syngeneic tumor-specific cytolytic specificity, after 17 weeks of continuous culture in T cell growth factor (TCGF; IL-2) we cloned the cells using TCGF in liquid suspension culture in microtiter plates (Baker et al., 1979). We hypothesized that we should obtain some clones with only alloreactivity and others with tumor-specific reactivity. Cells were seeded by limiting dilution at 0.3–0.1 cells/well, so that by Poisson statistics the probability that wells would be seeded with more than one cell was <0.05. Remarkably, the calculated plating efficiency ranged from 67 to 100%. Of 24 clones tested for cytolytic activity against allogeneic vs. syngeneic leukemia cells, 10 (42%) specifically only lysed the allogeneic targets, four clones (17%) lysed only the syngeneic targets, six clones (25%) lysed both allogeneic and syngeneic targets, and four (17%) were not cytolytic for either target. The cytolytic pattern of the clones remained constant over several weeks of culture, and to further prove clonality, one clone was selected and subcloned: all subclones demonstrated identical cytolytic activity. This was the first report that it was possible to derive true monoclonal cytolytic T cells. We concluded that, “*detailed studies of the phenotypic and functional characteristics of monospecific, homogeneous, cytolytic T lymphocytes will now be possible*” (Baker et al., 1979).

The methods detailed in this first paper regarding T cell cloning (Baker et al., 1979) were rapidly taken up and reproduced by everyone interested in generating their own antigen-specific T cell clones. Noteworthy among one of the first confirmatory reports using our methods to expand and grow large quantities of clonal progeny, was the creation of a clone cytolytic for a minor histocompatibility antigen (H-Y) by von Boehmer et al. (1979). Von Boehmer’s group immunized female mice with male H-Y^+^ splenocytes, then activated the female splenocytes by repetitive MLC *in vitro, *followed by growth in soft agar containing Con-A T cell supernatant (CATSUP) until microscopic colonies (20–30 cells) could be observed, picked, and expanded in lymphocyte conditioned medium (Ly-CM). Thus, the introduction of the growth-promoting properties of the Ly-CM were critical, in that they enabled the continuous propagation and expansion of the progeny so that the cells could be characterized and proved to be derived from a single cell. Also, when these investigators re-cloned the original cell line to prove clonality, they used our limiting dilution method rather than colony formation in soft agar. Limiting dilution cloning is much simpler and very efficient.

## MONOCLONAL ANTIBODIES AND HUMAN T CELL SURFACE MOLECULES (T3, T4, T8)

The other major advance that occurred during the 1970s was George Kohler’s and Cesar Milstein’s development of methods to create somatic cell hybrids (hybridomas) using mouse plasmacytoma cells and B cells from splenocytes from mice immunized with SRBCs, thereby generating monoclonal antibodies (MoAbs) with defined antigen specificity (Kohler and Milstein, 1975). This advance was truly revolutionary, because for the first time hybridomas could be generated and expanded to produce unlimited quantities of individual antibodies that could be used for a myriad of research purposes. In particular, the hybridoma technology brought the study of human immune responses into the forefront, because it was simple to immunize mice with human cells and molecules to produce specific MoAbs, whereas obviously, mouse cells and molecules were not immunogenic for mice. Not until almost a decade later were MoAbs raised against murine antigens, by immunizing either rats or hamsters to produce B cell fusion partners for murine plasmacytomas.

One of the first breakthroughs was from Reinherz et al. (1979b) who reported the separation of functional subsets of human T cells by a MoAb. Using a MoAb designated OKT4 raised against human peripheral T cells (Kung et al., 1979), Reinherz et al. (1979b) found that the MoAb reacted with ~ 60% of peripheral human T cells, while it was unreactive with human B cells, null cells and macrophages. Separation of human peripheral T cells into OKT4^+^ and OKT4^-^ subsets, followed by testing for proliferative responses to T cell mitogens and antigens, indicated that both subsets were responsive to T cell mitogens, but that most of the proliferative capacity of T cells resided in the OKT4^+^ population. Even more remarkable, after an MLC, most of the cytolytic activity was attributable to the OKT4^-^ subset, while the OKT4^+^ subset appeared to provide helper activity for cytolytic T lymphocyte (CTL) generation, as we had shown for TCGF (Baker et al., 1978). As noted by the authors, the OKT4 MoAb appeared to recognize the human T cell subset equivalent to that defined by murine Ly1 alloantisera reported by Kisielow et al. (1975) and independently by Cantor and Boyse, 1975). Additional experiments by Reinherz et al. (1979a) affirmed that the OKT4^+^ subset provided “help” for the generation of antibody forming cells (AFCs) from human B cells whereas the OKT4^-^ subset did not. However, the mechanism(s) whereby the helper T cells (Th) promoted both the generation of CTL and AFCs remained to be defined.

Monoclonal antibodies that recognized the reciprocal, cytolytic/suppressor subset, OKT5/8 (subsequently renamed CD8) was reported soon thereafter by Reinherz et al. (1980b). This MoAb was found to react with the human homolog of the murine determinant Ly2, recognized by the murine alloantisera. By using MoAbs, instead of alloantisera, and the new, very sensitive technique of flow cytometry (Ledbetter et al., 1980), Leonard Herzenberg’s group found the mouse Ly1 alloantigens are expressed on all T cells to varying amounts, so that a reciprocal marker for the mouse helper/inducer T cell subset, like human T4, was lacking. Actually, a MoAb that recognized the murine homolog of human T4 was only generated 4 years later by Dialynas et al. (1983) working with Frank Fitch and his group.

## HUMAN MONOCLONAL CYTOLYTIC T CELLS

In the interval, Reinherz’s group had already derived both T4^+^ and T5/8^+^ human alloreactive cytolytic T cell clones. By stimulating peripheral blood mononuclear cells (PBMCs) with an Epstein–Barr virus (EBV)-transformed B cell line in an MLC, T cell clones were obtained using both colony formation in soft agar and limiting dilution, followed by expansion in Ly-CM containing TCGF. They found that the T5/8^+^ clones were reactive against HLA-A and B antigens (MHC class I), while the T4^+^ clones were reactive against immune-associated (Ia) antigens (MHC class II; Meuer et al., 1982). Thus, whatever the nature of the T cell antigen receptor (TCR), these cloned T cells specifically recognized either the serologically defined HLA-encoded molecules or I-region encoded molecules on the surface of alloantigen-presenting cells, but not both. It is noteworthy that of fifteen T8^+^ clones tested, all exhibited a high level of cytotoxicity against the stimulating alloantigen, while only two of seven T4^+^ clones tested exhibited cytotoxicity. However, it is equally noteworthy that T4^+^ clones could become cytolytic, indicating that there was not a strict delineation between the functions of human T4^+^ vs. T8^+^ clones, such as helper vs. cytolytic that had been defined by studying T cell populations.

Of equal or even greater importance for understanding T cell antigen recognition was Reinherz’s report that the OKT3 MoAb blocked antigen-induced T cell proliferation (Reinherz et al., 1980c). By comparison with the T cell subset MoAbs, this MoAb recognized all peripheral T cells as well as ~10% of thymocytes with high immunoreactivity by flow cytometry. In this regard, reminiscent of Peter Nowell’s experiments showing that glucocorticoids suppressed phytohemagglutinin (PHA)-induced lymphocyte blastogenesis and mitosis only if added soon after PHA (Nowell, 1961), OKT3 was only maximally suppressive when added at the initiation of antigen stimulation. By comparison, several other MoAbs had no suppressive effects whatsoever, including OKT1, OKT4, OKT5/8, anti-Ia, and anti-beta-2-microglobulin. Reinherz interpreted their findings as: “*Both the appearance of this antigen in intrathymic ontogeny and its critical role in T cell function suggests that the T3 molecule is related to an important antigen recognition receptor*.”

In this regard, it is noteworthy that Reinherz’s group had used the reciprocal anti-T4 and anti-T5/8 MoAbs, together with anti-T3 to show that the majority of human thymocytes were positive for both T4 and T5/8 (i.e., “double positive”), while <10% of thymocytes were T3^+^, and these cells were only positive for either T4 or T5/8 expression, but not both (i.e., “single positive”; Reinherz et al., 1980a). It was not until 5 years later that the first murine T3 molecule was identified (van den Elsen et al., 1985), so that Reinherz’s findings were finally confirmed in the mouse. Of note, the First International Cluster of Differentiation (CD) Workshop nomenclature committee was so compelled by the wealth of data on T3, T4, and T8 that these molecules were named CD3, CD4, and CD8 accordingly^1^ (Bernard and Boumsell, 1984). These workshops were very important, because they allowed investigators to test their MoAbs to ascertain whether they were reactive with known CDs or whether new CDs should be designated. Today there more than 350 designated CD markers, and the list is still growing^2^.

## THE INTERLEUKINS

While these articles focused on human antigen-specific recognition by T cells, my team was focused on the antigen non-specific nature of the activities of soluble factors involved in the antigen-specific adaptive T cell response. When we submitted our findings describing the TCGF bioassay (Gillis et al., 1978), one of the reviewers asked how we could discriminate TCGF from macrophage-derived lymphocyte activating factor (LAF; Gery and Waksman, 1972). The LAF bioassay depended upon demonstrating enhanced proliferative activity of macrophage supernatants on murine thymocytes activated by PHA and cultured at high density (10^7^ cells/mL) for several days (Gery et al., 1972). Also, as already noted, it was well known that purified T cells were markedly less responsive to T cell mitogenic lectins than cell populations containing both lymphocytes and macrophages (Oppenheim et al., 1968). Accordingly, we tested for Con-A-induced TCGF production by purified T cells compared with unpurified splenocytes, and found that TCGF production was reduced by ~85%, as determined by the TCGF quantitative assay (Smith, 1980). Although adherent cells alone produced no detectable TCGF activity, reconstitution of purified T cells with small numbers of adherent cells completely restored TCGF production (Smith et al., 1980a). By comparison, thymocytes did not produce detectable TCGF.

These findings indicated that adherent cells and mature T cells must somehow cooperate upon mitogenic lectin stimulation to produce TCGF. Furthermore, they suggested that perhaps the limiting factor in thymocyte TCGF production was a relative deficiency of adherent cells. However, as shown by Reinherz, only ~10% of thymocytes were mature, and we found that only the cortisol-resistant thymocytes, which comprised ~10% of thymocytes, were capable of producing TCGF (Smith et al., 1979). Therefore, perhaps the majority of thymocytes, being immature simply could not produce TCGF. We still could not be sure which cell type actually produced TCGF, macrophage or mature T cell. Thus, in collaboration with Joost Oppenheim’s group, we tested his purified preparation of human macrophage-derived LAF and his purified preparation of human lymphocyte-derived mitogenic activity, and found that the LAF had no activity in the TCGF assay, whereas the lymphocyte-derived activity was positive in both the LAF assay and the TCGF bioassay. Also, our TCGF preparation scored positively in both the thymocyte LAF assay and the CTLL TCGF assay. We presented our findings together at the Second International Lymphokine Workshop that was held in Ermatingen, Switzerland, in May 1979, and for the first time it was appreciated by all investigators present that by using the TCGF bioassay it was possible to discriminate between monocyte/macrophage-derived LAF and lymphocyte-derived TCGF (Oppenheim et al., 1980). These findings electrified the conference and led to many late night discussions as to how to interpret the fact that LAF and TCGF were separable, at least functionally.

**FIGURE 1:**
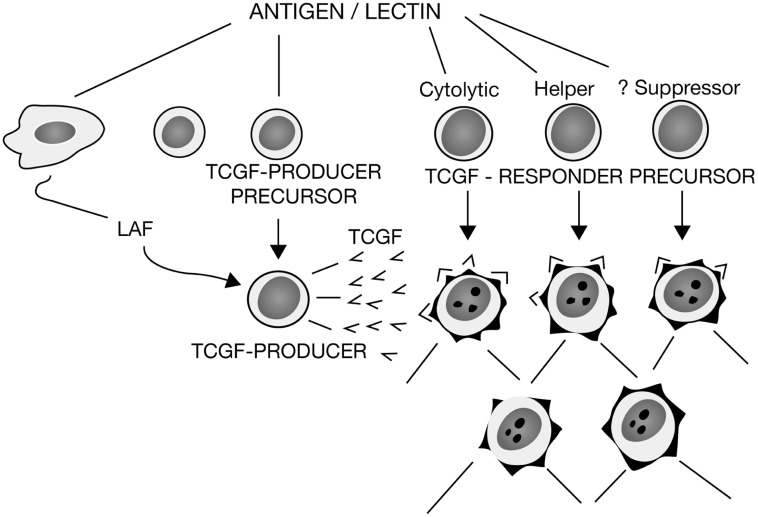
**A model for T cell activation**. Depicts the relationship between macrophage-derived LAF and T cell-derived TCGF, with the mitogenic activity of LAF due to its promotion of TCGF production, and the activity of TCGF to promote the proliferation of both cytolytic and helper T cell subsets, with suppressor T cell precursors still questionable. Redrawn from Smith (1980).

Even so, it was still unclear as to how LAF could be mitogenic for thymocytes and purified T cells, but not mitogenic for CTLL. Therefore, in additional experiments, we showed that purified LAF preparations prepared from lipopolysaccharide (LPS)-induced human PBMCs prompted cloned murine lymphoma cells to produce TCGF in a LAF-concentration-dependent manner (Smith et al., 1980b). Thus, it appeared that LAF promoted TCGF production and that both LAF and TCGF comprised “*a bimodal amplification system for the T cell immune response*.”**Moreover, because LAF was routinely produced from macrophages via stimulation by LPS, a common immunological adjuvant, these data indicated that this could explain how adjuvants like LPS functioned to markedly amplify immune responses. In still other experiments in collaboration with Oppenheim and Lawrence Lachman, using their purified human and murine LAF preparations respectively, we confirmed these findings and extended them by showing that glucocorticoids inhibited LAF production from macrophages, and consequently the LAF-induced proliferation of thymocytes, but did not suppress the TCGF-induced proliferation of thymocytes (Smith et al., 1980c). All of these data were included in a review that summarized our progress and proposed a new model for T cell activation that explained many of the experimental findings that had been enigmatic, as shown in **Figure 1** (Smith, 1980).

Accordingly, because LAF and TCGF were clearly distinguishable functionally by the TCGF assay, and because LAF was mitogenic for thymocytes and purified T cells because it enhanced T cell TCGF production, these data provided the scientific rationale for the interleukin nomenclature. Thus, subsequent to the Ermatingen Workshop, a proposal was circulated and signed by most of those investigators working in the field of antigen non-specific T cell proliferation and helper factors. It was proposed that LAF be renamed interleukin-1 (IL-1) because it worked upstream, and TCGF be named interleukin-2 (IL-2), because it was downstream of LAF activity (Letter, 1979). The term interleukin**was coined to designate that messages were passed between (inter)**leukocytes (leukin)*. *Like the complement field, we anticipated additional interleukins yet to be discovered. Now, in 2012 there are 37 interleukins, which constitutes the “modern” nomenclature for these newly discovered hormone-like molecules.

By comparison, identification of the molecule(s) responsible for antigen-specific helper and suppressor activities first described in the early 1970s (see Toward a molecular understanding of adaptive immunity: a chronology – part I) still had not progressed beyond their original descriptions, i.e., the removal of the activities by antigen-bound Sepharose and immunoaffinity columns of Ia alloantisera. As chairman of the session on antigen-specific factors, Marc Feldmann summarized the lack of progress in defining the biochemical natures of the various antigen-specific helper and suppressor factors at the Second International Lymphokine Workshop in 1979: “*All we can say at this moment is that there is probably a family of helper and suppressor factors*”**(Feldmann, 1980).

## THE FIRST INTERLEUKIN MOLECULE, IL-2

Although the new nomenclature in many ways simplified soluble antigen non-specific mitogenic factors by placing them into two categories, one macrophage-derived and the other lymphocyte-derived, the nomenclature was premature, because neither IL-1 nor IL-2 activities had been purified to homogeneity and ascribed to single, individual molecules. Thus, by 1980 it was clear to all in the field that the next step was purification.

Most investigators chose to attempt to identify and purify the molecules responsible for murine mitogenic activities (Watson, 1973; Di Sabato et al., 1975; Farrar et al., 1978; Shaw et al., 1978a,b). An illustrative report by James Watson in collaboration with Lucien Aarden, Jennifer Shaw, and Vermer Paetkau focused on the purification of 200 mL of supernatant from 10 Balb/c spleens (~10^9^ splenocytes) activated with Con-A for 18 h in media supplemented with 1% fetal calf serum (FCS; Watson et al., 1979). After ammonium sulfate precipitation of the proteins, the sample was subjected to molecular gel filtration, ion exchange chromatography, and isoelectric focusing (IEF). After each step, fractions were monitored for activity in three distinct assays; (1) T cell replacing activity for splenocyte AFCs, (2) Con-A-induced thymocyte mitogenesis-the LAF assay, and (3) induction of thymocyte alloantigen-specific CTL. These investigators found that fractionation of the Ly-CM by these methods yielded results that indicated “*the molecules responsible for biological activity in each assay system show identical behavior upon gel filtration, ion-exchange chromatography, and isoelectric focusing*” (Watson et al., 1979)*.*

By gel filtration, the activities corresponded in size to molecules from 30 to 40 kDa, and when applied to an ion-exchange column eluted with a salt gradient from 0.05 to 0.5 M ammonium acetate (pH 7.6), with identical activities in both the thymocyte LAF and AFC assays. By comparison, when monitored by IEF, there was considerable heterogeneity in pI in the broad range of pH 4–5. As already mentioned, both macrophage-derived mitogenic factors (i.e., LAF) and putative T cell-derived mitogenic factors (i.e., TCGF, Thymocyte stimulating factor, TSF, T cell replacing factor, TRF) could not be discriminated by these bioassays. Because the target cells used, i.e., thymocytes and splenocytes, were heterogeneous, when fractions of Ly-CM derived from heterogeneous producer cells containing both macrophages and lymphocytes (and both B cells and T cells), it was impossible to dissect the contributions of one cell and molecule vs. another, and whether the target cells of a given activity in turn released an additional activity that was actually detected by the assay. Thus, it was truly GIGO (garbage in, garbage out).

Also, the Ly-CM was produced in media containing 1% FCS. Accordingly, it is important to calculate that 1% = 1 g protein/dL = 10 mg/mL of FCS proteins in the Ly-CM. These investigators estimated that their cytokine activities were present in the Ly-CM at concentrations ~10^-^^9^ M, which at molecular sizes of ~35 kDa = 35 ng/mL. Therefore, the cytokine activities were produced in media with a million-fold excess of FCS proteins vs. cytokine proteins. In fact, as we were to learn subsequently, TCGF is active at concentrations in the pg/mL range, so that by using 1% FCS to make the Ly-CM, the FCS proteins were actually a billion-fold in excess of the cytokine proteins. This made the purification of the FCS proteins away from the cytokine proteins essentially impossible. Also, because the cytokine protein(s) were present in infinitesimal amounts, it meant that to be successful, one needed to begin with very large quantities of Ly-CM. If the cytokine was present at 150 pg/mL = 10 pM, then 200 mL of Ly-CM contained only 30 ng of cytokine protein. This was definitely not enough to quantify using any of the available protein assays, such as the colorimetric Bradford assay, which requires >1 μg protein (Bradford, 1976). Accordingly, even given 100% recovery of starting material, one would need to start with at least 7 L of Ly-CM, which would equate to 350 mouse spleens, not just 10.

By comparison with these efforts, we elected to focus on the purification and characterization of molecules with human TCGF activity. Having solved the problem of target cell heterogeneity by using murine CTLL clones, we could be confident that any effects observed were mediated by direct interaction of the lymphokine with the target T cells themselves. We decided to focus on human TCGF because ultimately we hoped to raise a murine MoAb reactive to the molecule(s), and because eventually we hoped to be able to use TCGF in the clinic. Others at the time favored the notion that a family of molecules would prove to have TCGF activity, based on the broad elution profiles from the molecular sieve and ion exchange columns and also from the multiple peaks observed after IEF. If true, when the molecules were separated, the activity discernable in the bioassay would be lost.

To approach the problem of serum proteins contaminating TCGF, we developed a system to produce TCGF in serum-free media from human PBMCs. To increase the amounts of starting material, we switched to human tonsil lymphocytes, because we could obtain ~10^9^ cells from each tonsil, which was equal to the number of lymphocytes in a liter of blood. Thus, it was possible to generate 1 L of PHA-induced Ly-CM from each tonsil, culturing the cells at 1 × 10^6^ cells/mL. Several liters of Ly-CM were pooled and concentrated >1,000-fold by filtration, then purified successively by gel filtration, IEF, and polyacrylamide gel electrophoresis (PAGE). We were able to show that the heterogeneity of charge that others had found examining murine Ly-CM could be eliminated by removal of sialic acid and by the inhibition of glycosylation. Thus, we could show that TCGF activity could be ascribed to a molecule with a single charge (p*I* = 8.2) and size (14–16,000 Mr), and that all of the apparent molecular heterogeneity was attributable to variable glycosylation and not due to multiple protein molecules with TCGF activity (Robb and Smith, 1981). Thus, for the first time, all of the TCGF biological activity could be ascribed to a single variably glycosylated protein.

## THE TCGF (IL-2) RECEPTOR

Having thus reduced the apparent molecular heterogeneity of TCGF activity to a single molecule, and knowing the biochemical characteristics of TCGF, i.e., it’s size and p*I*, we produced biosynthetically radiolabeled TCGF by culturing cells with amino acids tagged with radioisotopes, and then purified the radiolabeled TCGF using gel filtration and IEF, until we had a single radiolabeled band on SDS-PAGE detectable by fluorography (Robb et al., 1981). By monitoring the TCGF activity using the bioassay, and by measuring the protein concentration of unlabeled purified TCGF by amino acid analysis and dye binding, we were able to assign our preparations a specific activity, i.e., U/μg protein, and knowing the molecular size (15.5 kDa) we could calculate the molar concentration of both labeled and unlabeled TCGF (i.e., CPM/pmol). Thus, for the first time, we could determine that TCGF was active in the pM range. Ultimately, repeated determinations yielded a dose–response in the range of 1–100 pM with an EC_50_ = 5–10 pM.

Because our radiolabeled TCGF preparations were homogeneous and contained no other radiolabeled molecules, we proceeded with classical radiolabeled ligand-binding assays (Robb et al., 1981). Kinetic and equilibrium binding experiments revealed that unstimulated human PBMCs had detectable binding sites, on the order of ~200 sites/cell (lower limit of detection = 60 sites/cell), but that upon activation, either with mitogenic lectins or alloantigens, the number of detectable binding sites increased remarkably, ~50-fold, to ~10,000 sites/cell. Upon plotting the equilibrium binding data of human radiolabeled TCGF to human cells by the method of Scatchard (1949), we found a single class of high affinity binding sites, with an equilibrium dissociation constant (*K*_d_) ~5–10 pM, whereas murine activated T cells and CTLL, the *K*_d_ was ~20 pM. Of utmost importance, the concentrations of radiolabeled TCGF that bound to cells, and the concentrations of unlabeled TCGF that promoted T cell proliferation (EC_50_ = 1 U/mL = 5 pM) were essentially identical. Moreover, when several other growth factors and lymphokines were tested, only TCGF successfully competed for radiolabeled TCGF binding.

Soon after the publication of these data, I was contacted by Thomas Waldmann. Takashi Uchiyama from his group had raised a MoAb that only reacted with a human leukemia cell line that had been used for immunization of mice to produce hybridomas (Uchiyama et al., 1981a,b). They speculated that their MoAb might recognize the TCGF receptor, since it did not react with normal resting T cells, but did react after the T cells were activated via mitogenic lectins or alloantigens. The very first experiments were definitive, in that the MoAb, subsequently called anti-Tac (for activated T cell), competed for radiolabeled TCGF binding in a concentration-dependent manner (Leonard et al., 1982). Also, anti-Tac precipitated a single glycoprotein of ~55 kDa from radiolabeled cell surface molecules. Accordingly, these experiments described the first MoAb reactive with an interleukin receptor.

These findings were very significant, because for the first time they indicated that the immune system, like all other systems in the body, is under endogenous control via hormone molecules. Hormones and receptors had already been classically defined by physiologists in the late nineteenth century (Langley, 1878) and early twentieth century (Langley, 1905), as substances secreted by cells that act to elicit a characteristic physiological response at very low concentrations via interaction with high affinity with a cellular receptor. Thus, TCGF had all of the characteristics of a *bona fide* hormone, including stereospecificity, high affinity, and a finite number of binding sites that are consequently saturable.

Prior to these findings, the immune system was viewed as regulated entirely from without via environmental molecules (antigens), that when introduced were recognized by specific antigen receptors, which led to the proliferation and differentiation of the cells which then cleared the antigens. Thus, it was taught that the immune system was distinct and special, set apart from every other organ system, and was only aroused and regulated from a quiescent state by external forces, much like the nervous system senses changes in the environment, e.g., temperature, light, sound etc. Therefore, it was thought that once the system cleared the offending antigen, if there was no longer a driving external force, it returned to quiescence. Consequently, this dogma was overturned by finding that antigen-specific T cell clonal expansion is regulated by an endogenous hormone-receptor system like all of the other organ systems. It remained true that the introduction of antigen activates the immune system, but after antigen recognition there is an endogenous endocrine-like molecular mechanism that drives the proliferation and differentiation of the cells that actually mediate the antigen clearance.

The concept that an endocrine mechanism is responsible for immunoregulation, instead of solely being antigen-regulated, necessarily invoked a way to turn off the IL-2/IL-2R interaction. Of course, logic dictated that clearance of the antigen should result in the removal of the TCR-directed signals that control the expression of IL-2 and its receptors. However, to be termed a true hormonal system, endocrinologists required evidence for a hormone-induced negative feedback regulation of either hormone production or receptor expression, or both. Accordingly, these questions would require additional time and experimental approaches.

## THE MOLECULAR NATURE OF THE TCR COMPLEX

Having developed and propagated IL-2-dependent human T4^+^ and T8^+^ cytolytic T cell clones, Reinherz was in a unique position to identify the molecules responsible for T cell antigen recognition. Thus, in a seminal report, Reinherz and his group cracked the enigma of the molecular nature of the antigen recognition components of the TCR, and revealed the entire TCR complex for the first time (Meuer et al., 1983c). Operating under Burnet’s clonal selection theory of immunity, which led to the hypothesis that “*there must exist discriminative surface recognition structures that are unique to individual antigen-responsive T cell clones*,”**Reinherz’s group used one of their T8^+^ cytolytic T cell clones to immunize mice to produce murine hybridomas, and developed a screening strategy to select for clone-specific (clonotypic) MoAbs.

It was first important to determine whether the surface structures that the clone-specific MoAbs identified were actually involved in antigen recognition. Two such MoAbs were found to block both the specific cytotoxic function and antigen-induced proliferation of the immunizing T8^+^ T cell clone. Also noteworthy, the MoAbs enhanced the proliferation of the T cell clone in response to IL-2. Moreover, the surface molecules defined by the MoAbs were linked to the T3 structure, but in contrast to T3 (Mr ~20 kDa), they precipitated two associated glycoproteins of apparent molecular weights of 49 and 43 kDa. Reinherz and his group interpreted their results in an understated, more British, than American fashion: “*It is likely that the clonotypic MoAbs define variable regions of the human T cell antigen receptor *(on the T cell clone)* because they recognize clonotypic structures and inhibit antigen-specific function.*”

Subsequently, to determine whether analogous receptor molecules could be found on other T cell clones of differing antigen specificity, MoAbs were generated against a T4^+^ cytolytic T cell clone (Meuer et al., 1983a). Three MoAbs from ~600 hybridomas were selected, cloned and recloned by limiting dilution. Analysis of the molecular nature of the T4^+^ clone surface molecules recognized by all three antibodies yielded a 90 kDa molecule under non-reducing conditions, and two distinct bands, a ~51 kDa α-chain and 43 kDa β-chain under reducing conditions, thus very similar to the chains observed on the T8^+^ T cell clone. Also, in a fashion similar to the effects of the T8^+^ clone-specific MoAbs, the T4^+^ cytolytic clone-reactive MoAbs blocked alloantigen-specific cytolysis, as well as proliferation, and enhanced IL-2 induced proliferation. These findings supported the conclusions; “*that the basic subunit composition of the antigen receptors on cells derived from both the T4+ and T8+ human T cell subpopulations is similar. In contrast, recognition of class II or class I alloantigens by these subsets may be determined by the associative recognition structures T4 or T8, which are independent from Ti/T3*.”**Also, “*the wide distribution of the 20/25 kDa T3 glycoprotein and the ability of anti-T3 antibodies to inhibit antigen specific function of all clones suggest that T3 is a constant portion of the antigen receptor complex*.”

Consistent with this view, the Reinherz group also derived a series of T4^+^ clones reactive with specific protein antigens, ragweed antigen E (RWAGE), and tetanus toxoid (TT; Meuer et al., 1983b). Incubation of these clones with specific antigen together with autologous APCs and B cells resulted in the production of IgG. These T cell clones demonstrated a clear restriction for autologous class II MHC molecules. Immunization with one of the RAGWE-specific clones yielded one clonotypic MoAb from ~500 hybridomas. Biochemical analysis of immunoprecipitates showed a similar 90 kDa protein under non-reducing conditions and 52 and 41 kDa under reducing conditions, along with the associated T3 20/25 kDa molecules.

Accordingly, for the first time Reinherz revealed for everyone the complete TCR complex utilized by both T cell subsets to recognize and react with antigen. Also, using both flow cytometry and radiolabeled quantitative clone-specific MoAb binding analysis, they found that both the clonotypic (Ti) and T3 structures to be expressed at a similar level on the cell surface, ~30–40,000 molecules/cell, whereas the density of T4 and T8 expressed by the clones was ~120,000–175,000 binding sites/cell, a 3–6-fold excess (Meuer et al., 1983a). Other experiments showed that resting, freshly isolated T cells had equivalent densities of all three TCR complex structures, so that antigen or IL-2 activation was speculated to induce the enhanced levels of the accessory molecules T4 and T8.

Because T3 and clone-specific (Ti) structures appeared to be associated as they were expressed in equivalent densities and were co-modulated by their respective MoAbs, additional studies were performed to further explore this apparent non-covalent interaction. Having multiple T cell clones available, cell surface radiolabeled molecules could be immunoprecipitated with anti-T3 and analyzed by SDS-PAGE under reducing conditions (Reinherz et al., 1983a). In addition to T3 molecules of 20 and 25 kDa, two larger bands of ~ 49 and 43 kDa were observed from all clones tested. Also, two-dimensional SDS-PAGE analysis of the molecules precipitated with anti-T3 revealed two identical molecules with distinct p*I*s from the 25 kDa molecules and five distinct p*I*s from the 20 kDa molecules from all of the T cell clones tested, both T4^+^ and T8^+^, thereby confirming their invariant nature. By comparison, the 43 kDa molecules co-precipitated by anti-CD3 showed considerable variability by p*I* analysis, suggesting peptide heterogeneity. Peptide maps confirmed this heterogeneity of the 43 kDa β-chain molecules from several clones, and in addition, some variability of the 49 kDa α-chain molecules was suggested, consistent with the view that these chains could participate in antigen recognition.

The Reinherz group then reasoned that if the clone-specific MoAbs actually recognized the antigen-binding chains of the TCR, they might also mimic antigen, and activate the T cell clones, provided they were presented on a solid support (Meuer et al., 1983d). Thus, individual clone-specific MoAbs were conjugated to Sepharose beads. As controls, anti-T3, anti-T4, and anti-T8 MoAbs were also conjugated to Sepharose. When tested for their capacity to induce IL-2 production and proliferation as monitored by ^3^H-TdR the results were clear-cut. Only anti-T3 and the appropriate anti-Ti induced both IL-2 production and proliferation of both T4 and T8 cytolytic clones. Moreover, only soluble anti-T3 and the appropriate anti-Ti were capable of inhibiting IL-2 production and proliferation induced by the solid-phase MoAbs. Subsequently, they repeated these experiments using helper/inducer T4^+^ clones specific for soluble protein antigens, with identical results (Meuer et al., 1983b). As concluded by the Reinherz team, “*these results provide compelling evidence to support the notion that anti-Ti antibodies define the antigen *(recognition) *receptor structure on individual clones.*”

They also pointed out that the T4 and T8 surface structures, although critical for MHC-restricted CTL effector function, were unnecessary for solid-phase MoAb-induced IL-2 production and clonal proliferation. Their results also indicated that endogenous IL-2 production was inseparable from clonal proliferation, and it was clear that a single cell could, under physiological conditions, both produce and respond to its own IL-2. Accordingly, the molecules involved in antigen recognition and response in adaptive immunity were thus resolved and revealed.

Additional experiments focused on the biochemical characterization of the α-chains and β-chains reactive with the clone-specific MoAbs showed that the α subunits were more acidic than the β subunits by IEF, and more importantly, two-dimensional peptide maps indicated that the β-chains precipitated from two distinct clones were unique, but did share two peptides in common (Acuto et al., 1983b). By comparison, a similar analysis of α subunits from different clones were more related, but not identical. Therefore, both subunits appeared to contain both constant and variable domains, similar to antibodies.

**FIGURE 2:**
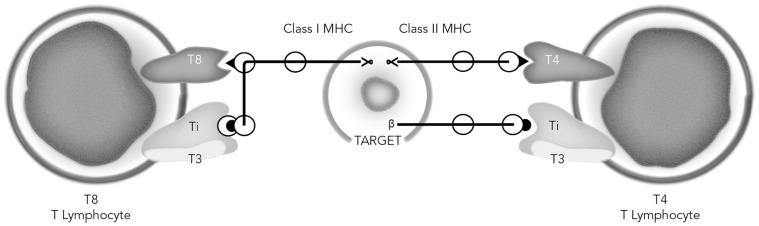
**Model of the human T cell receptor**. Each T lymphocyte displays two recognition units on its surface: the Ti-T3 complex and the associative recognition structure, T8 or T4. The T8 and T4 glycoproteins bind to non-polymorphic regions of class I and class II MHC gene products respectively. Note that the precise domains of class I and class II that the T cells recognize are unknown and assigned in the figure only for purposes of illustration. The Ti molecules, unique to each T cell recognize specific antigen in the context of a polymorphic MHC gene product, while the T3 molecules, common to all mature T cells, are involved in signaling the cell interior. Redrawn from Reinherz et al. (1983b).

Accordingly, these initial molecular characterizations indicated some similarities between the αβ antigen recognition elements of the TCR and antibodies. However, there the similarities ended, in that an invariant component like T3 involved in signaling had not been found to be associated with surface immunoglobulin on B cells. Also, B cells did not express accessory molecules similar to T4 and T8 as did T cells. Moreover, the data accumulated by Reinherz’s group indicated that restriction of T cell antigen recognition to MHC class I or class II correlated with the expression of T8 or T4 respectively. Also, it was noteworthy that the T8/class I and T4/class II interaction did not necessarily dictate T cell function of cytolysis vs. help, since T4/class II-restricted T cell clones could obviously kill appropriate target cells. Finally, all of these data indicated that only one 90 kDa heterodimeric TCR antigen receptor could recognize alloantigens, and only one receptor could recognize foreign protein antigens, such as RWAGE and TT + MHC encoded molecules simultaneously. All of these findings were summarized in January, 1983 (Reinherz et al., 1983b) as shown in **Figure 2**.

A few months before Reinherz’s first report on the molecular nature of the entire TCR complex (Meuer et al., 1983c), Allison et al. (1982) reported that they had generated a MoAb reactive with a surface antigen of a T cell lymphoma. Their data indicated that “*the MoAb was highly specific for the T lymphoma cells used for the immunizations, and did not react with a panel of other spontaneous or X-ray-induced or chemically-induced lymphomas*.”**Of interest, on biochemical analysis of surface radiolabeled proteins, the MoAb precipitated “*a glycoprotein composed of disulfide bonded subunits of 39,000 and 41,000 m.w.*”**In addition, Allison’s group used a technique of “diagonal” two-dimensional gel electrophoresis combining an SDS-PAGE under non-reducing conditions followed by re-electrophoresis under reducing conditions. Because most surface molecules are not disulfide linked, most of the proteins migrate on a diagonal under these conditions. However, the disulfide linked proteins reactive with the MoAb migrated off of the diagonal, because when reduced, the two chains separated into their lower sizes.

Operating under the hypothesis that the tumor-specific antigen recognized by their MoAb might be part of a surface molecule normally expressed specifically by T cells, the Allison group subjected several different types of cells to two-dimensional non-reducing/reducing SDS-PAGE. They found that purified T cells and thymocytes had proteins migrating off of the diagonal in positions very similar to the MoAb immunoprecipitated proteins from the T cell lymphoma. By comparison, bone marrow cells (mostly myeloid and erythroid precursors) and B cells did not express detectable proteins migrating in this manner. The Allison team speculated that their MoAb might recognize the “*T cell equivalent of the B cell idiotype*,”**but because they had raised their MoAb against a tumor cell, they had no way to test for antigen recognition or immunological reactivity, in contrast to Reinherz, who had used normal functional IL-2-dependent T cell clones.

Then, a few months after Reinherz’s first seminal report, another group led by Philippa Marrack reported the generation of a MoAb reactive with an antigen-specific murine somatic T cell hybrid that they had produced by fusing immune Balb/c splenocytes with an azaguanine-resistant thymoma cell line (Haskins et al., 1983). This “T cell hybridoma,” DO-11.10, was selected for its capacity to produce IL-2 when stimulated with specific antigen, chicken ovalbumin (cOVA), in the presence of APCs of the appropriate MHC type. Then, splenocytes from a DO-11.10-immunized mouse were used to make a B cell hybridoma, which was screened for the capacity to inhibit the production of IL-2 by cOVA-stimulated T cell hybrids. Supernatants from one cloned hybridoma, KJ1-26, was selected for study. The KJ1-26 MoAb-containing supernatants selectively inhibited IL-2 production only from the immunizing T cell hybridoma, and not from six other antigen-specific T cell hybridomas. In addition, this MoAb specifically inhibited binding of the DO-11.10 hybridoma to antigen-pulsed APCs, suggesting that the KJ1-26 MoAb might recognize a surface structure that bound antigen.

Identification of the molecules reactive with the KJ1-26 MoAb revealed that it immunoprecipitated a molecule of ~ 80–90 kDa by SDS-PAGE under non-reducing conditions and two molecules of 40–44 kDa under reducing conditions. Similar to the Allison group’s experiments using “diagonal” non-reducing/reducing SDS-PAGE, the KJ1-26 immunoprecipitates revealed a spot that was below the diagonal, consistent with it’s disulfide-bonded structure. Accordingly, like the Allison group, the Marrack group had generated a MoAb that reacted with an antigen of similar biochemical characteristics expressed by a T cell hybridoma, and in addition, they had data showing that the MoAb blocked antigen-specific MHC-restricted IL-2 production. However, neither the Allison nor the Marrack groups had data revealing any other T cell functions such as proliferation or cytolysis, as they were dealing with cells that proliferated autonomously. Also, there were no data regarding the T3 signaling components of the TCR complex, nor the nature of the MHC restricting structures T4 and T8. Their reports on the biochemical characteristics of the putative antigen recognition elements in these mouse lymphoma cells and hybridoma cells thus confirmed Reinherz’s findings regarding the antigen recognition elements of the human TCR complex, but they did not describe the entire murine TCR complex on both T4-class II restricted and T8-class I-restricted T cell clones of both cytolytic as well as inducer/helper functional phenotypes, as had Reinherz’s reports.

Once the data from all three groups became known to one another, they cooperated in their next reports, which were published simultaneously in the same journal. The Reinherz group reported the generation of an additional MoAb reactive with a human T cell leukemia/lymphoma cell line, which was T3^+^, T4^+^, and T8^+^ (Acuto et al., 1983a). Three MoAbs that reacted specifically only with this T cell tumor were selected for study. These MoAbs resembled the clone-specific MoAbs raised against the normal T cell clones, in that they did not react with normal T cells, B cells, macrophages, granulocytes, or RBCs. Moreover, each of these tumor-specific MoAbs co-modulated clone-specific surface structures, as well as T3, but did not change the surface expression of T4, T8, or T11. SDS-PAGE analysis of immunoprecipitates under non-reducing conditions revealed a single band at 94 kDa, and two bands under reducing conditions (53 and 44 kDa). Peptide maps of the putative α and β-chains showed that they were distinct molecules, and that they both shared peptide fragments in common with chains immunoprecipitated from normal T cell clones, thereby indicating that all of the clonotypic MoAbs precipitated chains that shared at least one peptide fragment, presumed constant domains, as well as clone-specific peptides, presumed variable domains. Also, using “diagonal” two-dimensional SDS-PAGE the Reinherz group substantiated their earlier reports regarding the T cell phenotype during thymic maturation (Reinherz et al., 1980a; Reinherz and Schlossman, 1980) by showing that the clone-specific TCR recognition molecules appear during intrathymic ontogeny in parallel with surface T3 expression, so that cells pass from triple negative (T3^-^T4^-^T8^-^) to double positive (T3^-^T4^+^T8^+^), to T3/Ti^+^ and single positive for either T4 or T8. Thus, the clonotypic antigen recognition elements are intimately linked to T3 expression during ontogeny, thereby providing a structural basis for the group’s previously reported observation that immunological competence is acquired only among the population of thymocytes that express surface T3 (Umiel et al., 1982). In this regard, it is noteworthy that only this very minor subset of thymocytes (<10%), which also are cortisone resistant, were found responsive to LAF (IL-1), and thus capable of inducing proliferation in the LAF (IL-1) thymocyte bioassay because they could produce TCGF (IL-2).

The Marrack group reported data on a second MoAb, this one reactive with a T cell hybridoma that produced IL-2 in response to a class I alloantigen (Kappler et al., 1983), their first MoAb being specific for a T cell hybrid reactive with an MHC class II-restricted cOVA antigen. This new MoAb, designated KJ12-98, suppressed IL-2 production by the class I-stimulated T cell hybrid, and in addition, when bound to Sepharose beads, stimulated IL-2 production, as Reinherz had shown for his clone-specific MoAbs. Also, similar to all of the previous reports this MoAb precipitated a disulfide linked heterodimer, ~85 kDa, which resolved into two subunits of 40–43 kDa. Subsequently, Samelson et al. (1983) reported two MoAbs reactive with a class II-restricted T cell hybridoma that responded to a pigeon cytochrome *C* peptide by producing IL-2. Like the MoAbs of the Marrack group, these MoAbs inhibited clone-specific antigen-induced IL-2 production, and precipitated disulfide-linked dimers comprised of 45–50 kDa molecules.

By comparison, McIntyre and Allison (1983) used their MoAb to isolate the reactive antigen from their T cell lymphoma cell line, which they then used to immunize a rabbit to generate a polyclonal antiserum. This antiserum precipitated disulfide-linked dimers from both normal thymocytes and peripheral T cells, but not non-T cells. Peptide maps indicated that the molecules from the different cell sources shared common subunits, as well as having non-homologous peptide fragments. However, they still did not have data indicating that either their MoAb or their antiserum reacted with a structure capable of recognizing antigens.

Accordingly, by the close of 1983 the enigmatic TCR complex had been revealed to all in the immunological community. From the chronology of the reports that appeared during this year, it is clear that the Reinherz group was first, complete and correct, as time would prove. By comparison, those working in the mouse necessarily followed Reinherz’s lead, thereby partially confirming his findings, but because they lacked identification of murine T3 and T4, they could not describe all of molecules of the entire TCR complex, and consequently they could not trace the expression of the recognition, accessory, or signaling elements during T cell ontogeny as did Reinherz. Moreover, because they chose to work with continuously proliferating T cell lymphomas and hybridomas instead of using normal IL-2-dependent T cell clones, they could not examine the function of the TCR complex in T cell activation, proliferation and any differentiated function such as cytolysis, beyond showing that their MoAbs interfered with antigen-induced IL-2 production.

## THE IL-2 cDNA, IL-2 GENE, IL-2 MoAbs, AND PURE HOMOGENEOUS IL-2

The year 1983 was also an important year for IL-2. Tadatsugu Taniguchi’s group took advantage of the rapid, specific and quantitative IL-2 bioassay to identify a cDNA encoding human IL-2 activity (Taniguchi et al., 1983). mRNA was isolated from the JURKAT human T leukemia cell line that had been found to produce IL-2 upon mitogenic lectin stimulation (Kaplan et al., 1975; Gillis and Watson, 1980). Using methods of hybrid selection of mRNA and translation in *Xenopus laevis *oocytes, followed by assay for TCGF activity using the CTLL-2 cells, a cDNA was identified that possessed TCGF activity. The cDNA specified 153 amino acids and a 20 amino acid hydrophobic leader signal sequence so that the mature secreted protein contained 133 residues, yielding a calculated molecular size of 15,420.5 Da, almost identical to the molecular size we estimated by SDS-PAGE of both native and radiolabeled TCGF (IL-2; Robb et al., 1981; Robb and Smith, 1981). Once the cDNA encoding IL-2 had been identified, both Taniguchi, as well as ourselves, isolated genomic clones encoding the entire IL-2 gene (Fugita et al., 1983; Holbrook et al., 1984). The IL-2 gene spans 8 kb and is organized into four exons.

Although these genetic studies were informative as to the primary structure of the IL-2 molecule, and would eventually permit studies on the TCR complex regulation of IL-2 gene expression, they did not immediately lead to the availability of large amounts of pure IL-2 for additional biochemical, biological, and immunological studies. However, we had developed several MoAbs reactive with IL-2, which we used as immunoabsorbants to purify milligram quantities of homogeneous IL-2 protein (Smith et al., 1983). Analysis of immunoaffinity purified IL-2 indicated that it eluted as a single peak from a reverse-phase high pressure liquid chromatography (HPLC), and migrated as a single size (15.5 kDa) on silver stained SDS-PAGE. Proof that there were no other contaminating proteins in the immunoaffinity purified preparations was obtained by N-terminal amino acid sequence analysis, which identified a single N-terminus, and the first 15 residues. Because this amino acid sequence was identical to that predicted from the IL-2 cDNA nucleotide base sequence, the data indicated agreement between the predicted cDNA and actual amino acid sequence analysis, and thus proving that a single molecule mediated IL-2 biological activity.

In addition to proving useful for the immunoaffinity purification of IL-2, the IL-2 MoAbs effectively neutralized both human and murine IL-2 activity, but were non-reactive with rat-derived IL-2. In addition, the neutralization of IL-2 could be competitively antagonized by an excess of IL-2. Moreover, the concentrations of IL-2 MoAbs that neutralized IL-2 in a 24 h bioassay were identical to the MoAb concentrations that block radiolabeled IL-2 equilibrium binding, which came to steady state within 15 min. These experimental results essentially proved that the MoAb neutralizing capacity depended upon IL-2 binding by the MoAbs, and not due to non-specific interference with cellular metabolism in the bioassay, a problem that had misled other investigators attempting to generate IL-2-specific MoAbs (Gillis and Henney, 1981; Stadler et al., 1982).

Additional experiments exploring the immunoaffinity adsorption characteristics of four distinct IL-2 MoAbs indicated that the single most important parameter is the association rate, and the temperature-dependence of both the association and dissociation rates (Budd and Smith, 1986b). Thus, as the association rate increases directly with temperature, we found that the most efficient immunoaffinity adsorption occurred at 37°C, while the washing and elution steps were best performed at 4°C. Moreover, the efficiency of immunoaffinity adsorption of separate MoAbs varied according to their association rates. We also used the IL-2 MoAbs to create the first immunoassay for an interleukin (Budd and Smith, 1986a). For these experiments radiolabeled IL-2 and radiolabeled IL-2 MoAbs were used to determine the reaction kinetics at each stage of the immunoassay. Noteworthy was the finding that reaction rates are retarded remarkably when performed in the solid-phase vs. solution, and are more rapid at 37°C than either 20°C or 4°C. These advances were paradigmatic for the uses of MoAbs raised against all of the interleukins that followed.

## cDNA CLONES ENCODING T CELL-SPECIFIC MEMBRANE PROTEINS

All of the data accumulated by Reinherz on the molecular nature of the TCR complex indicated that human T cells expressed surface molecules distinct from B cells that were involved in antigen recognition, as well as the T3 signaling molecules and the T4 and T8 accessory molecules associated with MHC restriction. The data accumulated on the αβ antigen recognition chains indicated that they were of a different size compared with heavy and light chains of antibodies, but like antibodies, the TCR chains did appear to have both constant and variable regions.

Thus, TakMak’s group took these data into consideration to search for the elusive TCR antigen recognition molecules (Yanagi et al., 1984). They screened for cDNA clones of mRNAs that were expressed either exclusively or preferentially in T cells, in contrast with others who had searched for Ig molecules on both B cells and T cells (Binz and Wigzell, 1975). Given the T cell-specific expression of T3, T4, and T8, they constructed a cDNA library from mRNA extracted from the human leukemia cell line MOLT3, which they found to express T3, T4, and T8 (Nagasawa and Mak, 1982). Of 10,000 cDNA clones screened by differential hybridization to mRNA from MOLT3 cells vs. a human B cell lymphoma, four were selected for further study, which were found to identify a single mRNA of 1.3 kb by northern blot analysis from MOLT3 that was not detected in the B cell line. Of several human leukemia cell lines screened, only T cell leukemias were positive, as well as normal human thymocytes and peripheral T cells, together with a mouse T cell leukemia cell line.

To determine the size of the protein encoded by their cDNA they used hybrid selection with mRNA from MOLT3 cells, followed by *in vitro *translation and analysis by SDS-PAGE, which yielded a single protein of ~30 kDa. The nucleotide sequence of one of the cDNA clones revealed a long open-reading frame that predicted a 34,938 kDa protein, two possible sites for N-linked glycosylation, a hydrophobic leader sequence at the N-terminus, and a hydrophobic region near the C-terminus resembling a membrane anchor. In addition, the predicted protein resembled human and murine Ig light chains, especially in the relative locations of the cysteine residues, as well as extensive homology over the entire length of the Ig variable, joining and constant regions. These results were very tantalizing, but there was no way to test for antigen recognition or any other function because they were dealing with lymphoma cells. Moreover, even if this represented the cDNA encoding an antigen recognition component of the TCR complex, it represented only one of the two chains. Thus, the Mak group could only conclude that, “*The nature and function of this protein are unknown; it may be similar to the known T cell-specific antigens *(T3, T4, T8),* or to the α- or β-subunits of the recently identified T-cell receptors*” (Yanagi et al., 1984).

Following this report, in the same journal issue Hedrick et al. (1984a) reported their work on a presumptive murine TCR antigen recognition structure. Also operating from a hypothesis that the T cell antigen recognition elements should be specifically expressed by T cells and not by B cells, this team crafted a strategy of constructing ^32^P-labeled cDNAs of the membrane-bound polysomal RNA fraction of murine antigen-specific T cell hybridomas, previously described by Samelson et al. (1983), assuming that mRNAs encoding a membrane molecule would be found on membrane-bound polysomes. They then subtracted these cDNAs by an excess of RNAs from B cell polysomes, to render the radiolabeled cDNA probes T cell-specific. T cell cDNA homologous to B cell RNA was removed by fractionation on hydroxyapatite, which binds double-stranded nucleotides. The resulting B cell-subtracted ^32^P-cDNA was used to probe a cDNA library constructed with mRNA from another B cell subtracted T cell cDNA preparation. From 5,000 clones screened, 30 were selected and used for further study. Seven clones hybridized with mRNA considered large enough to code for a TCR recognition element. They next hypothesized that the TCR recognition elements should undergo genetic rearrangements to create the diversity of antigen recognition in a manner similar to Ig genes. Thus, they constructed probes from each of the seven clones and hybridized them in Southern blots with genomic DNA from liver cells from the mouse strains used and genomic DNA from the parent T cell lymphoma used to construct the T cell hybridomas, as well as genomic DNA from the antigen-specific T cell hybridoma. Only one clone of the seven was consistent with DNA rearrangements, which were found only in the T cell lymphoma and hybridomas.

The nucleotide sequence of their longest cDNA clone predicted a leader sequence of 19 residues, a 98 residue region with similarity to murine Ig variable regions, a 16 amino acid J**region, followed by a constant-like region, also similar to murine Ig constant regions. The similarity in sequence between the predicted T cell cDNA and Ig was so pronounced that it was conjectured that it could conceivably explain the reported cross-reactivity of B cell anti-idiotypic antisera with T cells (Binz and Wigzell, 1975). Finally, there was a predicted hydrophobic membrane-spanning region near the C-terminus. Thus, the authors concluded that “*it must almost certainly play a part in antigen recognition by T cells*.”**In support of this contention, they cited unpublished work which indicated that antisera raised against peptide fragments predicted by their cDNA clone significantly inhibited the antigen-dependent release of IL-2 by antigen-stimulated T cell hybridomas (Hedrick et al., 1984b).

Another way to approach the primary sequence of a TCR antigen recognition molecule was to purify the α and/or β-chains and perform N-terminal amino acid sequence analysis. Thus, the Reinherz group chose to use their clone-specific MoAbs reactive with their REX T-leukemia cell line because they could grow the requisite number of cells more easily than their IL-2-dependent T cell clones. Lysates from 5 × 10^10^ cells were immunoaffinity purified using their clonotypic MoAb. Focusing on the smaller β-chain (~ 41–44 kDa), 20–50 μg of protein could be obtained from ~ 5 × 10^10^ cells. N-terminal sequencing reproducibly yielded the first 12 residues, minus the first amino acid. Comparison of this sequence with other proteins in the data base yielded a weak homology with a human Ig λ V-region.

The Reinherz report (Acuto et al., 1984) was submitted for publication just 3 days before the cDNA sequences were reported. Thus, Reinherz added a note in galley proof:

*“Additional NH_2_-terminal sequencing of Ti β subunit has identified amino acid residues 12, 14, and 16-20 and thus yields the following sequence: X-Val-Ile-Gln-Ser-Pro-Arg-His-Glu-Val-Thr-Glu-X-Gly-X-Glu-Val-Thr-Leu-Arg. These 17 amino acids are identical to 17 residues within the predicted protein sequence of the YT35 cDNA clone defined by* Yanagi et al. (1984)............. *In addition, the high degree of nucleotide homology between the 3′ end of the YT35 human cDNA clone and murine cDNA clones* (Hedrick et al., 1984b)...... *suggests that the mouse equivalent of the Ti β gene has (also) been isolated”* (Acuto et al., 1984).

Thus, by two different methods, protein and nucleotide sequencing, the primary structure of the first TCR complex antigen recognition chain became known. In addition, the two methods validated one another.

## TURNING ANTIGEN-SPECIFIC ACTIVATION INTO ANTIGEN NON-SPECIFIC ACTION

From all of these data, it appeared that although triggering of the TCR complex initiates T cell proliferative clonal expansion, there was a heretofore-unknown molecular mechanism that actually mediated the complex intracellular biochemical reactions that perform the necessary molecular events that culminate in DNA replication and cytokinesis. In this regard, one of the puzzling aspects of T cell proliferation after mitogenic or antigenic stimulation was the transient nature of the proliferative response. As noted by Nowell (1960), after the addition of a mitogenic lectin such as PHA, there ensues an initial lag of 48–72 h, then a rapid burst of proliferation, followed by a gradual cessation of proliferative activity and ultimately a cessation of proliferation. Once it was appreciated that the TCR complex is responsible for IL-2 production, and as well, once it became possible to monitor the TCR complex induction of IL-2R expression, these critical parameters regulating the T cell proliferative response could be examined. It appeared that the TCR complex *per se* could not promote DNA replication and cytokinesis.

From our initial studies of IL-2 production after mitogenic lectin triggering, we already knew that there was a burst of IL-2 secretion by the activated cells, followed by a gradual cessation of IL-2 release, and the disappearance of detectable IL-2 concentrations over the succeeding several days (Gillis et al., 1978). Therefore, this phenomenon alone could explain the transience of the proliferative response. However, once it became possible to monitor IL-2R expression, both quantitatively with the radiolabeled IL-2 binding assay, and qualitatively by flow cytometry using the IL-2R MoAb, Cantrell and Smith (1983) performed a series of experiments on the biological importance of IL-2R expression. Using IL-2 rendered homogeneous by MoAb immunoaffinity purification, biosynthetically radiolabeled IL-2 also immunoaffinity purified with the IL-2-reactive MoAbs, and IL-2R-reactive MoAbs, the growth characteristics of PHA-activated T cells were determined and correlated with IL-2R expression for the first time. IL-2Rs appear asynchronously in lectin-activated human peripheral T cell populations and precede the onset of DNA synthesis monitored by ^3^H-TdR incorporation, as well as by propidium iodide (PI) quantitative DNA staining and flow cytometry. Moreover, upon removal of the activating lectin at the peak of IL-2R expression after 72 h of culture, IL-2R levels do not persist. Rather, a time-related and IL-2 independent decay of IL-2Rs occurs that is rapidly reversible by re-stimulation with lectin. As IL-2R appearance and disappearance is mirrored and followed by the proliferative rate of the cells within the population, our results indicated that the lectin-induced IL-2R densities were of primary importance, along with adequate IL-2 concentrations, in determining the extent of T cell clonal expansion, and consequently by extrapolation, the tempo, magnitude, and duration of the resultant T cell immune response (Cantrell and Smith, 1983).

Although these studies were definitive with regard to IL-2 and IL-2Rs, because we had performed the experiments using a mitogenic lectin as the activating signal, we could not be sure whether they represented the true physiology of the adaptive T cell immune response. Thus, it was a natural extension of these studies for us to collaborate with Ellis Reinherz’s group, once he had established normal human T cell clones, and had generated clone-specific MoAbs. Accordingly, combining Reinherz’s unique cellular and molecular TCR complex reagents with our unique IL-2 and IL-2R molecular reagents, for the first time it was possible to perform reductionist science on the molecular nature of the T cell adaptive immune response, thereby moving from the micrometer to the nanometer level in immunology (Meuer et al., 1984).

To investigate the relationship between the TCR complex and IL-2-mediated T cell growth, we used three antigen-specific human T cell clones and a series of clone-specific MoAbs in conjunction with homogeneous immunoaffinity-purified human IL-2, as well as MoAbs reactive with both IL-2 and the IL-2R. First, we established that two human cytolytic T cell clones (T4^+^ and T8^+^), and a protein antigen-specific helper/inducer T4^+^ clone, proliferated *in vitro* in response to homogeneous purified IL-2, thus consistent with our studies that the active ingredient in Ly-CM was actually IL-2. Second, this IL-2-dependent proliferative response was essentially completely abrogated by the IL-2R MoAb, and was reduced from 67 to 75% by the neutralizing IL-2-reactive MoAb. In contrast, MoAbs reactive with the constant portion of the human Ia antigen (MHC class II), that like the IL-2R appears on activated human T cells, did not diminish IL-2-mediated clonal proliferation (Meuer et al., 1984).

As observed previously with Ly-CM containing IL-2, we found that activation using soluble clone-specific MoAbs enhanced by 2–3-fold ^3^H-TdR incorporation of each clone in response to pure homogeneous IL-2. As with IL-2 alone, this increased ^3^H-TdR incorporation initiated by the anti-clonotype MoAbs was completely abrogated by the IL-2R-reactive MoAb and reduced by two-third by the MoAb that could neutralize IL-2. Since anti-clonotypic MoAbs did not promote proliferation without exogenous IL-2, we surmised that the soluble MoAbs may trigger the expression of IL-2Rs, but not IL-2 production. This hypothesis was tested by flow cytometry. The expression of IL-2Rs was found to be stimulated sixfold by stimulation with anti-clonotypic MoAb, and as well, by specific antigen + APCs. Accordingly, these data confirmed and extended our previous results using PHA, to the physiologic reagents of anti-clonotypic MoAb, and specific Ag + APCs.

**FIGURE 3:**
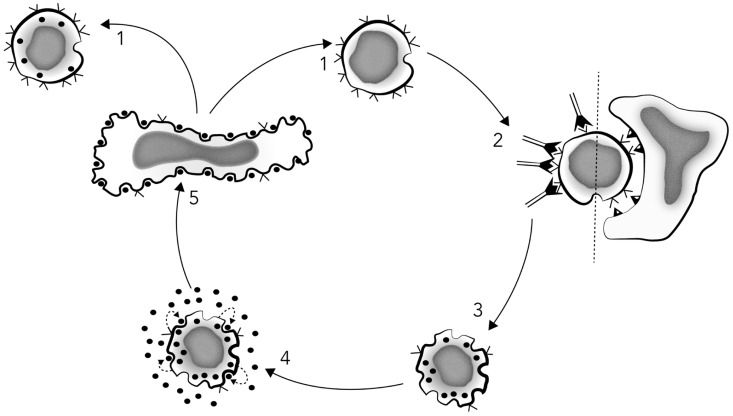
**Schematic model of T cell proliferation mediated by an IL-2-dependent autocrine mechanism**. *Stage 1*: resting T cells or T cell clones express few or no IL-2 receptors, while reciprocally displaying a maximal number of antigen receptors (V). *Stage 2*: T3-Ti triggering by antigen/MHC-restricting element or surface bound anti-T cell receptor antibodies results in T3-Ti modulation, thus reducing the number of surface antigen receptors and rapidly inducing surface IL-2 receptor expression. *Stage 3*: This event appears to occur prior to IL-2 secretion itself because IL-2 receptor expression can be observed 2–4 h after T3-Ti triggering, whereas IL-2 is not detectable in culture supernatants until at least 10–12 h later. Stage 3 can also be achieved with anti-Ti or anti-T3 monoclonal antibodies in soluble form. *Stage 4*: activation via Ti-T3 leads to production and secretion of endogenous IL-2 (•) and subsequent binding to its own IL-2 receptors. *Stage 5*: once a critical density of occupied IL-2 receptors is achieved, DNA synthesis and mitosis occur. Finally, in the absence of additional antigenic stimulation, there is re-expression of the surface T3-Ti complex (stage 1). Redrawn from Meuer et al. (1984).

Further experiments comparing activation with soluble vs. solid-phase anti-clonotypic MoAbs, and either cellular alloantigens or Ag + APCs, showed that soluble anti-clonotypic MoAbs promoted IL-2R expression but not IL-2 production, whereas the same anti-clonotypic MoAbs bound to Sepharose beads promoted both the expression of IL-2Rs and IL-2 production, as did both alloantigens and Ag + APCs. Thus, “*The present construct clearly implies that T cell proliferation is mediated through an autocrine network in which antigen-receptor triggering leads to IL-2 production, IL-2R expression, IL-2 release, and subsequent IL-2R occupancy, which ultimately promotes cell division” *(Meuer et al., 1984), as depicted in **Figure 3**.

During the course of these experiments, we noted that IL-2R expression after TCR complex activation occurred asynchronously among the individual cells within a T cell population. Moreover, examination at the single cell level using the cytofluorograph and IL-2R MoAbs, a broad range of IL-2Rs/cell was observed, spanning as much as three orders of magnitude. Accordingly, we wondered whether differences in IL-2R density made any difference to the IL-2-dependent proliferative response. In this regard, a purview of the literature revealed that all cells, both prokaryotes as well as eukaryotes, proliferate asynchronously, with some cells proliferating faster than others, even among cloned cell populations. Thus, we wondered whether this universal characteristic pattern of cell growth could be related to the density of growth factor receptors as well as the concentration of the growth factor.

For the first time in any cell system, we had accumulated the necessary molecular and cellular reagents to explore this fundamental aspect of cellular proliferation. Of utmost importance, a T cell population could be synchronized into the G_0_–G_1_ phase of the cell cycle by removal of IL-2, and then maximal levels of IL-2Rs could be induced by re-stimulation with mitogenic lectins. Upon exposure to IL-2, the kinetics of IL-2-dependent cell cycle progression could then be followed and analyzed in relationship to IL-2R density and distribution among the cells within the population (Cantrell and Smith, 1984).

When the density of IL-2Rs is monitored by the cytofluorograph with IL-2R MoAbs, and plotted on a log_10_ scale, like all surface molecules, a log-normal distribution of IL-2Rs is observed. Since the proportion of occupied receptors is dependent on the IL-2 concentration, with a log-normal distribution of IL2Rs/cell, at any given IL-2 concentration the absolute number of occupied IL-2Rs should vary according to the receptor density of each cell. It follows that if the number of occupied IL-2Rs is critical for the decision to replicate DNA, a G_0_–G_1-_synchronized cell population with a log-normal distribution of IL-2Rs would be expected to enter the proliferative phases of the cell cycle asynchronously, as a function of the IL-2 concentration, which was readily observed. The IL-2 concentration-dependency was most easily discerned by selecting a single time interval after the addition of IL-2: a typical sigmoid log-dose–response curve resulted whether the response was monitored by ^3^H-TdR incorporation or PI staining, which indicated that the magnitude of ^3^H-TdR incorporation reflected the proportion of cells that had left G_0_–G_1_ and entered the proliferative phases of the cell cycle (Cantrell and Smith, 1984).

In other experiments, the duration of the IL-2/IL-2R interaction was found to be a critical determinant of cell cycle progression, so that there appeared to be an interplay between at least two variables, IL-2 concentration and the duration of the IL-2/IL-2R interaction, such that the number of cells responsive to a suboptimal IL-2 concentration could be increased by lengthening the exposure period. These observations suggested that the absolute number of IL-2/IL-2R interactions occurring during the G_1_ phase of the cell cycle was critical for the decision of a cell to divide. A series of experiments varying the IL-2 concentration, the IL-2R density or the duration that these two molecules interacted proved this hypothesis, and for the first time showed that cellular division is a deterministic and not a probabilistic phenomenon. Moreover, the decision is quite simple and depends upon only four variables: (1) the affinity of the IL-2/IL-2 interaction, which dictates (2) the IL-2 concentrations necessary to saturate the IL-2Rs, but (3) the IL-2R density/cell and (4) the duration of the IL-2/IL-2R interaction are equally important (Cantrell and Smith, 1984).

## CONCLUSION

In just 5 years, the understanding of adaptive immunity underwent a sea-change, from a science focused on populations of cells to one focused on individual cells and molecules for the first time. No longer was the TCR complex, both structurally and functionally, an enigma. Gone were the notions that T cells recognized antigens via Ig molecules, although the antigen recognition structures were found to be members of what came to be known as the Ig superfamily. Moreover, the TCR complex became defined as comprised of antigen recognition structures of α- and β-chains, as well as accessory molecules, T4 and T8, that facilitated recognition of antigens “in the context” of MHC-encoded molecules, and which were found to be intimately involved in T cell ontogeny. Moreover, the TCR complex included the T3 signaling molecules, which were expressed by all T cells. Also gone were antigen-specific factors, as T cells were not found to secrete their antigen recognition structures, as do B cells. Moreover, the MHC-encoded molecules also were shown not to be TCRs, and not to be secreted as helper or suppressor “factors.” Rather, the genetic control of the T cell immune response was found to be involved with antigen presentation via MHC-encoded molecules, which T cells recognize via only one receptor, not two. In addition, T cells were found to mediate their antigen-specific responses via signaling the expression of antigen non-specific molecules, the cytokines or interleukins, and their receptors. Consequently, the immune response could no longer be considered a unique, passive system that only functioned in response to external signals from the environment. Like all of the other bodily systems, the immune system became appreciated for the first time to be regulated by endogenous hormone-like molecules via specific hormone-like receptors. These changes in our perception of how the adaptive immune system is structured and how it functions led immediately to a new vista of experimental possibilities, one where the exploration of a myriad of new molecules and biochemical signaling pathways have made immunology one of the most interesting and rapidly expanding areas of biology, and one where it is now on its way to contribute in a major way to twenty-first century medicine. Moreover, to launch this quest, the torch was passed to a new generation of immunologists armed with cloned cells, cloned antibodies and cloned genes, techniques that were transformative for the new science of molecular immunology.

## Conflict of Interest Statement

The author declares that the research was conducted in the absence of any commercial or financial relationships that could be construed as a potential conflict of interest.

## Footnotes

1 http://www.uniprot.org/docs/cdlist

2 http://en.wikipedia.org/wiki/Cluster_of_differentiation

## Abbreviations

AFC: antibody forming cell
anti-Tac: MoAb reactive with IL-2Rα chain
APC: antigen-presenting cell
CATSUP: con-A T cell supernatant
CD: cluster of differentiation
CTL: cytotoxic T lymphocyte
CTLL: cytotoxic T lymphocyte line
EBV: Epstein–Barr virus
HPLC: high pressure liquid chromatography
Ia: immune-associated antigen (MHC class II)
IEF: isoelectric focusing
IL: interleukin
LAF: lymphocyte activating factor (IL-1)
LPS: lipopolysaccharide
Ly-CM: lymphocyte conditioned media
MLC: mixed leucocyte culture
PBMC: peripheral blood mononuclear cell
PHA: phytohemagglutinin
RWAGE: ragweed antigen E
SDS-PAGE: SDS polyacrylamide gel electrophoresis
T3: CD3
T4: CD4
T8: CD8
TCGF: T cell growth factor
TCR: T cell antigen receptor complex
Ti: clonotypic TCR
TRF: T cell replacing factor
TSF: T cell stimulatory factor
TT: tetanus toxoid.
